# Biodistribution of Intra-Arterial and Intravenous Delivery of Human Umbilical Cord Mesenchymal Stem Cell-Derived Extracellular Vesicles in a Rat Model to Guide Delivery Strategies for Diabetes Therapies

**DOI:** 10.3390/ph15050595

**Published:** 2022-05-12

**Authors:** Junfeng Li, Hirotake Komatsu, Erasmus K. Poku, Tove Olafsen, Kelly X. Huang, Lina A. Huang, Junie Chea, Nicole Bowles, Betty Chang, Jeffrey Rawson, Jiangling Peng, Anna M. Wu, John E. Shively, Fouad R. Kandeel

**Affiliations:** 1 Department of Translational Research & Cellular Therapeutics, Beckman Research Institute of the City of Hope, Duarte, CA 91010, USA; hkomatsu@coh.org (H.K.); kellyhuang1220@g.ucla.edu (K.X.H.); lahuang@exeter.edu (L.A.H.); jrawson@coh.org (J.R.); jianglingpeng@gdut.edu.cn (J.P.); 2 Department of Radiopharmacy, Beckman Research Institute of the City of Hope, Duarte, CA 91010, USA; kpoku@coh.org (E.K.P.); jchea@coh.org (J.C.); nbowles@coh.org (N.B.); 3 Department of Cancer Molecular Imaging and Therapy, Beckman Research Institute of the City of Hope, Duarte, CA 91010, USA; tolafsen@coh.org (T.O.); betchang@coh.org (B.C.); awu@coh.org (A.M.W.); jshively@coh.org (J.E.S.)

**Keywords:** extracellular vesicles (EVs), umbilical cord mesenchymal stem cell (UCMSC), diabetes, I-124, positron emission tomography (PET), intravenous (I.V.) administration, intra-arterial (I.A.) administration, biodistribution

## Abstract

Umbilical cord mesenchymal stem cell-derived extracellular vesicles (UC-MSC-EVs) have become an emerging strategy for treating various autoimmune and metabolic disorders, particularly diabetes. Delivery of UC-MSC-EVs is essential to ensure optimal efficacy of UC-MSC-EVs. To develop safe and superior EVs-based delivery strategies, we explored nuclear techniques including positron emission tomography (PET) to evaluate the delivery of UC-MSC-EVs in vivo. In this study, human UC-MSC-EVs were first successfully tagged with I-124 to permit PET determination. Intravenous (I.V.) and intra-arterial (I.A.) administration routes of [^124^I]I-UC-MSC-EVs were compared and evaluated by in vivo PET-CT imaging and ex vivo biodistribution in a non-diabetic Lewis (LEW) rat model. For I.A. administration, [^124^I]I-UC-MSC-EVs were directly infused into the pancreatic parenchyma via the celiac artery. PET imaging revealed that the predominant uptake occurred in the liver for both injection routes, and further imaging characterized clearance patterns of [^124^I]I-UC-MSC-EVs. For biodistribution, the uptake (%ID/gram) in the spleen was significantly higher for I.V. administration compared to I.A. administration (1.95 ± 0.03 and 0.43 ± 0.07, respectively). Importantly, the pancreas displayed similar uptake levels between the two modalities (0.20 ± 0.06 for I.V. and 0.24 ± 0.03 for I.A.). Therefore, our initial data revealed that both routes had similar delivery efficiency for [^124^I]I-UC-MSC-EVs except in the spleen and liver, considering that higher spleen uptake could enhance immunomodulatory application of UC-MSC-EVs. These findings could guide the development of safe and efficacious delivery strategies for UC-MSC-EVs in diabetes therapies, in which a minimally invasive I.V. approach would serve as a better delivery strategy. Further confirmation studies are ongoing.

## 1. Introduction

Extracellular vesicles (EVs) have recently demonstrated tremendous potential as a therapeutic alternative to cell-based therapy for a wide range of diseases. These nanosized, membrane-bound vesicles are integral modulators of intercellular communication and contain a variety of cellular components, such as cytokines, growth factors, signaling lipids, mRNA, and regulatory miRNAs, depending on the cell origin [1,2]. Compared to cell-based therapy, EVs provide significant clinical advantages including low immunogenic and tumorigenic properties, efficient cellular uptake, homing capabilities, easier quantification and storage, and the ability to escape degradation to provide long-term releasing effects [3]. With such broad regulatory functions, EVs have emerged as a promising treatment strategy for various clinical applications.

A growing number of studies have focused on harnessing the capacity of EVs to recapitulate the therapeutic effects of mesenchymal stem cells (MSC). MSC-derived EVs (MSC-EVs) efficiently transfer therapeutic agents to enhance proliferation, attenuate apoptosis, activate autophagy, and regulate immune reactivity [4,5,6,7,8,9]. In preclinical studies, MSC-EVs are capable of accelerating tissue regeneration and inducing angiogenesis for osteochondral defects [10], skeletal muscle injury [11], and myocardial ischemia and reperfusion [5,12]. Their therapeutic capacity has also extended to repairing liver fibrosis [13], promoting the recovery of acute renal injury [14], enhancing cutaneous wound healing [15], improving pulmonary hypertension [16], reducing amyloid-beta deposition for Alzheimer’s disease [8], and suppressing cell growth and migration for cancers [17,18].

Due to their immunomodulatory and metabolic functions, MSC-EVs have become an emerging strategy for treating various autoimmune and metabolic disorders, particularly type 1 diabetes mellitus (T1DM) and type 2 diabetes mellitus (T2DM). T1DM is characterized by beta-cell dysfunction and death from autoreactive immune cells, reduced insulin production, and hyperglycemia. Meanwhile, T2DM involves peripheral insulin resistance and a reduction in pancreatic beta-cell mass. Diabetes mellitus often leads to severe complications, such as chronic refractory wounds, diabetic nephropathy, diabetic neuropathy, retinopathy, and increased risk of stroke. Preclinical studies have demonstrated MSC-EVs are capable of effectively regenerating pancreatic beta-cell mass, ameliorating autoimmune reaction, restoring insulin production, and preventing disease onset in T1DM murine models [19,20,21]. Additionally, MSC-EVs promoted hepatic glucose and lipid metabolism, reversed insulin resistance, and reduced beta-cell destruction in rat models of T2DM [22,23]. These studies shed light on the potential of MSC-EVs to modulate and remedy diabetes pathogenesis. MSC-EVs have also shown to ameliorate comorbidities by suppressing renal cell apoptosis for diabetic nephropathy [24], improving functional recovery for diabetic peripheral neuropathy [25], promoting neurorestoration following stroke in T2DM [26], reducing inflammatory reaction for diabetic retinopathy [27], and accelerating diabetic wound healing [28,29,30]. Although MSC-EVs have never been clinically studied for diabetic patients in the United States to our knowledge, preclinical evaluation of the therapeutic potential of MSC-EVs would greatly accelerate the translation to clinical therapies.

Umbilical cord-derived MSCs are attracting substantial research attention as a promising source of MSC-derived EVs (UC-MSC-EVs) for diabetes therapy. In vivo studies have revealed that various types of cell-derived phospholipid bilayer enclosed vesicles, namely EVs, displayed different distributions. Currently, it is unclear whether intravenous (I.V.) or intra-arterial (I.A.) administration of UC-MSC-EVs provides more efficient delivery. Therefore, comparing the administration routes is a crucial step to translate UC-MSC-EVs into practice. Positron emission tomography (PET) is well established as an imaging modality, offering high sensitivity to monitor the target radiolabeled PET isotopes in various tissues in vivo. In this study, we utilized PET imaging strategies to directly compare delivery routes for human UC-MSC-EVs. By administering radioiodinated human UC-MSC-EVs ([^124^I]I-UC-MSC-EVs) in a rat model, we aim to elucidate whether I.V. or I.A. administration is better to enhance EV-based delivery. Considering the incredible potential of human UC-MSC-EVs as a versatile therapeutic strategy, these findings may guide the development of safe and efficacious delivering strategies for clinical treatments.

## 2. Results

### 2.1. Radioiodination and Stability of UC-MSC-EVs

**Figure 1:**
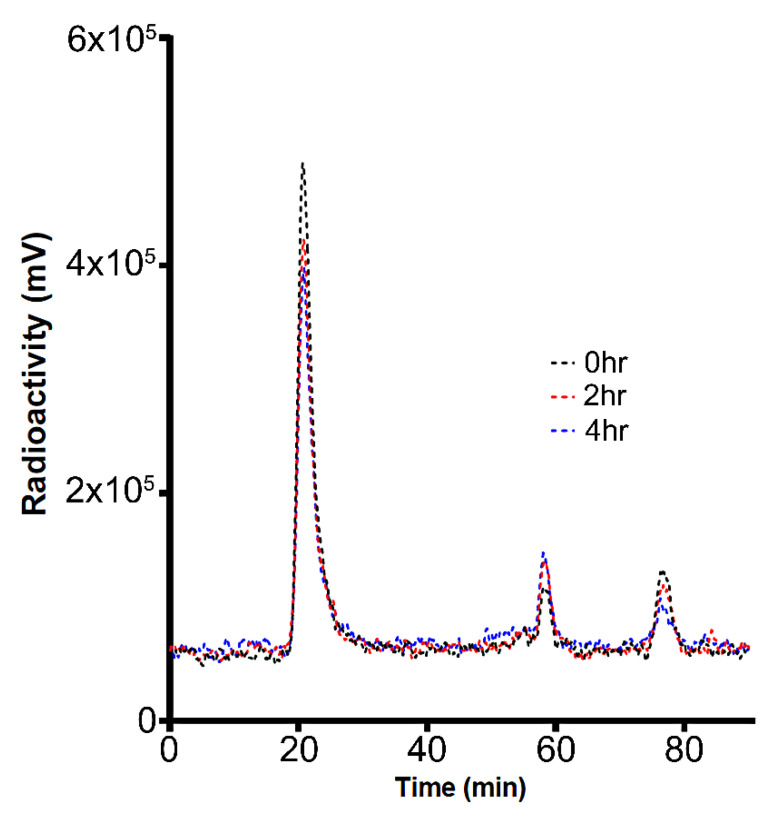
Radiochemical stability test for [^124^I]I-UC-MSC-EVs. The samples were incubated for 0, 2, and 4 h to assess radiochemical stability. The results indicated that the radiochemical purity of [^124^I]I-UC-MSC-EVs was >95% (T = 0, 2, and 4 h), and only two minor peaks were observed.

^124^I possesses an intermediate half-life of 4.18 days, making it one of the optimal radioisotopes for the evaluation of biomolecules, including EVs. Radioiodination of human UC-MSC-EVs with ^124^I-sodium iodide was successfully performed using the direct IODOGEN methodology. Purification of [^124^I]I-UC-MSC-EVs was carried out via Superdex 200 chromatography, achieving high radiochemical purity (>99%) by ITLC. Due to the lengthy process required for I.A. injections, radiolysis of [^124^I]I-UC-MSC-EVs was of concern, but further analysis revealed that [^124^I]I-UC-MSC-EVs displayed high stability (>95%) even after 4 h (Figure 1). Only two minor peaks were found (total <5%). These findings indicate that the same batch of [^124^I]I-UC-MSC-EVs may service 2–4 rats for PET-CT scanning.

### 2.2. I.V. Administration and PET-CT Small Animal Imaging

**Figure 2:**
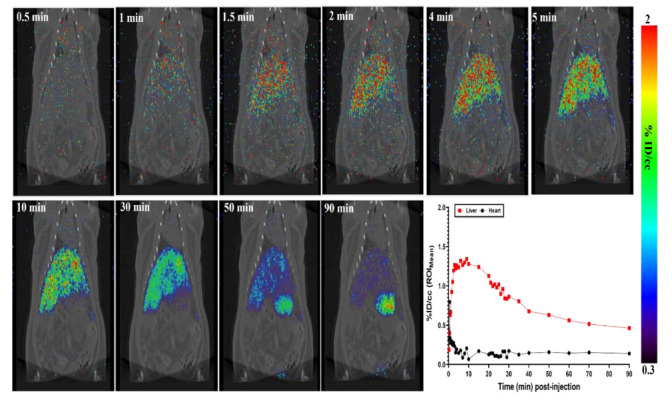
MicroPET imaging of I.V. administered [^124^I]I-UC-MSC-EVs in male rats. The representative MRP PET/CT serial images were shown (**top and left bottom**) at 0.5 min, 1 min, 1.5 min, 2 min, 4 min, 5 min, 10 min, 30 min, 50 min, and 90 min post injection. MicroPET imaging provided clear visualizations of [^124^I]I-UC-MSC-EVs accumulation and clearance in the organs from 0 to 90 min. Tissue time-activity curve of the liver and heart were also shown (**right bottom**).

MicroPET imaging of [^124^I]I-UC-MSC-EVs was conducted for rats that underwent the I.V. injection route. Briefly, male rats (350–500 g) were anesthetized with 2–4% isoflurane in oxygen and received approximately 8 MBq of [^124^I]I-UC-MSC-EVs in 1% HSA with PBS via the tail for I.V. injections. 0–90 min dynamic body PET scan (48 frames: 15 s × 16; 30 s × 6; 60 s × 8; 300 s × 1; 60 s × 10; 300 s × 2; 600 s × 5) followed by 1 min computed tomography (CT) scan were performed. The representative dynamic images from 0 to 90 min and regions of interest (ROIs) are shown in Figure 2. MicroPET imaging provided clear visualizations of the accumulation and clearance of [^124^I]I-UC-MSC-EVs in the organs/tissues.

Early dynamic PET images did not display high uptake in all organs within 1 min after injection. Heart uptake ROIs reached the highest levels (%ID/cc of ~0.45) at 1 min post-injection, followed by rapid clearance. The highest liver uptake was observed at 10 min post-injection (%ID/cc of ~1.40), and gradually decreased by ~60% at the end of the scan. Although clearance of [^124^I]I-UC-MSC-EVs was observed, it was difficult to characterize clearance patterns through PET imaging for the stomach, spleen, pancreas, and small intestine, due to the proximity of the organs to each other in the rat. As a result, we utilized ex vivo biodistribution analysis to validate uptake levels from PET imaging. No significant uptake in the lung and pancreas was observed.

We conducted a separate independent microPET-CT study (~8 MBq [^124^I]I-UC-MSC-EVs) to evaluate uptake levels in the male rat head. The findings indicated that low uptake was observed in the whole brain throughout the 90 min dynamic PET scan post-injection (microPET results not shown).

### 2.3. I.A. Administration and PET-CT Small Animal Imaging

I.A. administration of ~ 8 MBq [^124^I]I-UC-MSC-EVs was successfully achieved in one surgical procedure. [^124^I]I-UC-MSC-EVs were infused into the pancreatic parenchyma of the rat body and tail through the celiac artery after blockage of the splenic artery, common hepatic artery, and left gastric artery. After the injection was completed, all clamps on the arteries were released, and the abdominal incision was closed. The rats subsequently underwent a 70 min dynamic body PET scan using 17 frames (60 s × 10; 300 s × 2; 600 s × 5) followed by a 1 min CT scan. For the I.A. injection, it was necessary to wait until hemostasis was achieved and to suture the wound closed. In order to compare the same time points for biodistribution analyses between the I.A. and I.V. injection routes, rats in the I.A. injection group underwent 70 min PET scans after a 20 min delay post-injection.

**Figure 3:**
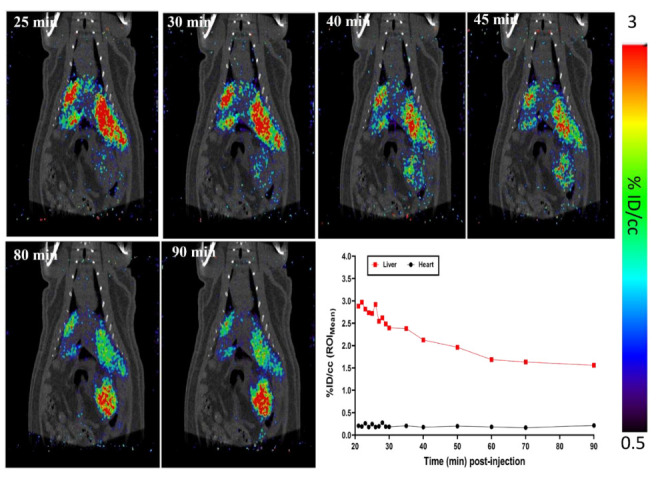
MicroPET imaging of I.A. administered [^124^I]I-UC-MSC-EVs in male rats. The representative MRP PET/CT serial images were taken (**top and left bottom**) at 25 min, 30 min, 40 min, 45 min, 80 min, and 90 min post-injection. Tissue time-activity curve of the liver and heart were also shown (**right bottom**).

The microPET images and ROIs were shown in Figure 3. PET scans for rats in the I.A. group displayed similar patterns as PET images for rats in the I.V. group. Both I.V and I.A. administration methods showed predominant liver distribution, with the I.A. group having greatest uptake at around 25–30 min post-injection (5–10 min after the initial PET scan). The time-activity curves (TACs) showed that liver uptake for the I.A. group was ~1.7 times higher compared to corresponding time points for the I.V. group. Importantly, the pancreas did not display high accumulation early in PET scanning. Other organs displayed uptake levels similar to the uptake levels for the I.V. injection group. Furthermore, clearance of [^124^I]I-UC-MSC-EVs from the liver was observed during the middle and end of the PET scan, but it again proved difficult to clarify clearance patterns. We therefore analyzed the biodistribution of [^124^I]I-UC-MSC-EVs to validate accumulation in organs.

### 2.4. Biodistribution Analysis of the I.V. Injection Group and the I.A. Injection Group

**Figure 4:**
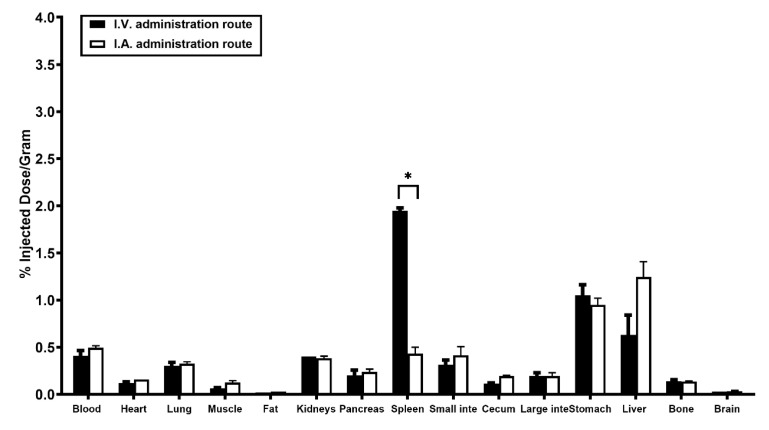
Comparative biodistribution of [^124^I]I-UC-MSC-EVs for the I.V. group vs. the I.A. group after PET-CT scanning (96–98 min post injection). Rats were injected with ~8 MBq of [^124^I]I-UC-MSC-EVs, and tissue biodistribution analyses were performed. * *p* <0.01.

Biodistribution analyses were performed following PET-CT imaging for both administration routes (Figure 4 and Table 1). Results from both groups indicated low overall uptake levels (%ID/g) in the blood, brain, heart, lung, small intestine, large intestine, muscle, kidneys, and bone. In the pancreas, both I.V. and I.A. administration routes demonstrated comparable accumulation levels of 0.20 ± 0.06 %ID/g and 0.24 ± 0.03 %ID/g, respectively.

The liver, which was the organ of predominant uptake, displayed accumulation levels consistent with the TAC results in both groups: 0.63 ± 0.21 %ID/g (I.V.) and 1.25 ± 0.16 %ID/g (I.A.). Uptake levels in the stomach were similar in the I.V. injection route (1.05 ± 0.11 %ID/g) and the I.A. administration route (0.95 ± 0.07 %ID/g), which was due to radio-deiodination of [^124^I]I-UC-MSC-EVs. The spleen displayed significantly higher uptake levels in the I.V. group compared to the I.A. group (1.95 ± 0.03 %ID/g vs. 0.43 ± 0.07 %ID/g, respectively). Furthermore, biodistribution results clarified that the region of high uptake towards the left lower area of the liver shown in the PET images was from the spleen (1.62 ± 0.07 %ID/organ) and stomach (2.43 ± 0.07 %ID/organ) in the I.V. group. For the I.A. group, the area of high uptake was from the stomach (~2.86%ID/organ), as the spleen had low accumulation levels (~0.95 %ID/gram).

## 3. Discussion

Although the method of delivery for biological molecules is important for optimizing the efficacy of clinical therapies, characterizing and quantifying delivery methods has been challenging. PET-CT is an optimal approach that combines the high sensitivity and quantification of PET with computed tomography (CT) scanning to provide anatomic data for co-registration. By allowing for non-invasive tracking of administered molecules. PET-CT imaging plays a key role in evaluating drug delivery in preclinical in vivo optimization cycles prior to validation in humans using the same techniques.

Due to their therapeutic potential, EVs have been characterized in preclinical trials. Although both I.V. and I.A. administration routes are efficient strategies in clinical applications, there have been no direct comparisons of these administration routes for UC-MSC-EVs. Theoretically, I.A. administration has the advantage of selective delivery to the pancreas but may increase the risk of occlusion or embolization. Meanwhile, I.V. administration is less invasive but may lead to sequestration in other organs, such as the spleen and lung, and thereby reduce EV delivery to the pancreas and cause off-target effects [31,32]. A previous comparison of I.V. and I.A. delivery of bone marrow mononuclear cells for acute ischemic stroke found no differences between the two modalities [33]. However, different EVs derived from cell types could exhibit varying characteristics, cellular targets, and therapeutic outcomes, so it is important to evaluate the potential of I.V. versus I.A. administration of UC-MSC-EVs. In the present study, human UC-MSC-EVs were tagged with a radioisotope (I-124) and underwent combined PET-CT imaging and biodistribution, which allowed for direct and sensitive monitoring of UC-MSC-EVs in vivo. This approach may guide further development of safe and efficacious delivery strategies for future clinical trials.

### 3.1. Surgical Procedures for I.A. Injection of [^124^I]I-UC-MSC-EVs

**Figure 5:**
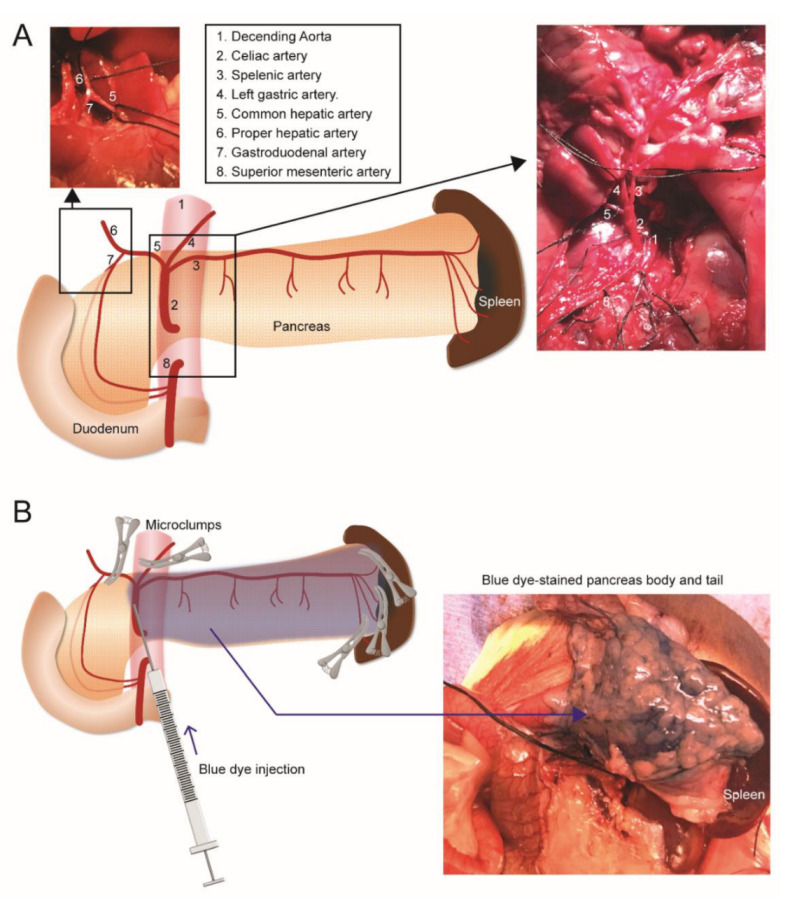
Intra-arterial injection procedure. (**A**) Anatomy of arteries around pancreas. The schema illustrates the description of the arteries, and two photographs show the dissected key arteries. Arteries were looped with black silk sutures. (**B**) Injection procedure. To specifically distribute the injected solution (blue dye in the preliminary tests or [^124^I]I-UC-MSC-EVs for PET imaging), common hepatic artery, left gastric artery, and terminal branches of splenic arteries were clumped. A needle was cannulated into the Celiac artery to inject the solution. Microclumps were released after the injection was completed.

[^124^I]I-UC-MSC-EVs were directly infused into the pancreatic parenchyma of the rat body and tail through arterial flow after blocking nearby arteries in the pancreas (Figure 5A, see Materials and Methods for more details). There were several major challenges in performing I.A. injections and subsequent imaging. Firstly, to perform precise surgical procedures, we used large rats (350–500 g). However, the large size of the rats precluded the ability to evaluate the tracer kinetic in the whole rat body within a single image, due to the small transaxial field of view (FOV) of 12 cm of the microPET imaging system. Instead, a separate scan was required to obtain whole body PET imaging. Therefore, the pilot studies separated imaging of the rats into two independent studies, including the head PET scan and the body PET scan. The low uptake of [^124^I]I-UC-MSC-EVs in the head observed in the PET images was confirmed by biodistribution analysis, and our subsequent PET studies were therefore focused on the rat body. Secondly, the surgical procedures required technical expertise. In preliminary trials, we confirmed the feasibility of the surgical procedure and distribution of the injected solution using methylene blue dye injection for non-survival surgeries (Figure 5B, see Materials and Methods for more details). Thirdly, avoiding radio-contamination of the tissues near the I.A. injection site proved challenging, particularly due to compound leakage from the high pressure-arterial flow. Accordingly, a small gauze adjacent to the injection site was pre-placed to absorb the leaked radioactive solution. After the injection was completed and hemostasis was achieved, radioactivity of the gauze was measured, and only non-radio-contaminated rats were employed for PET-CT imaging. Fourthly, the procedure was quite invasive since it involved a large laparotomy in a survival surgery. Therefore, health status during the 70 min imaging period was another concern. To determine health status, we performed a pilot study, which confirmed that breathing rates were stable under general anesthesia for 2 h after the I.A. surgery procedure, demonstrating the feasibility of the surgical procedure being followed by image acquisition. In fact, the complexity of the surgical procedures was attributed to the small animal experimental model. In the clinical setting, I.A. is safely performed as a catheter-assisted procedure, in which the catheter is generally inserted through the femoral artery of the patient to the splenic artery for pancreas-selective distribution of the solution.

### 3.2. Radio-Deiodination In Vivo

The low stability of the carbon-iodine bond in [^124^I] analogues may cause substantial radio-deiodination in vivo, thus leading to the formation of free radioiodine that will rapidly accumulation in the thyroid and stomach. Therefore, all rats were pretreated with 10 drops of saturated KI per 100 mL of drinking water for 24 h before injection of [^124^I]I-UC-MSC-EVs. PET imaging results indicate that rat thyroid uptake of radioiodine was successfully blocked. Additionally, uptake of radioiodine in the stomach is typically blocked by gastric lavage with potassium perchlorate in PBS 30 min before injection. However, to avoid complicating the surgery procedure of the I.A. group, we did not pre-treat the stomach. As a result, accumulation of radioiodine was observed in the stomach during the middle and later stages of PET imaging.

### 3.3. PET-CT Small Animal Imaging and Biodistribution

In PET imaging, the predominant uptake of [^124^I]I-UC-MSC-EVs occurred in the liver for both injection routes during the early and middle stages. Clearance of [^124^I]I-UC-MSC-EVs was observed from the liver during the middle and late stages. However, it was difficult to characterize clearance patterns for the stomach, spleen, pancreas, and small intestine, since these organs were located close together in the rat. Therefore, we utilized biodistribution analysis to validate uptake levels from PET imaging. Splenic uptake for both modalities displayed significant differences, which may be explained by the pathways of the administration routes. For the I.V. injection route, [^124^I]I-UC-MSC-EVs were likely filtered out once they reached the spleen. However, splenic uptake levels following I.A. injection depended on which arteries were blocked during the surgical procedure. After releasing the vessel clamps, the [^124^I]I-UC-MSC-EVs were likely filtered by the liver, so higher uptake levels were observed in the spleen for I.V. administration compared to I.A. administration, and vice versa for the liver. Uptake levels (%ID/gram) in the spleen were significantly different between the two injection routes, whereas uptake levels in the liver were not, due to the extremely small amount of [^124^I]I-UC-MSC-EVs compared to the entirety of the liver and the spleen, and the greater weight of the liver compared to the spleen (~20–24 g for liver vs. 0.8–1.2 g for spleen) in a big rat. Overall, the high sensitivity of the nuclear imaging technique provided a reliable and quantitative evaluation of pancreas uptake in both administration routes, and demonstrated, importantly, that both I.V. and I.A. injection resulted in similar uptake levels in the pancreas. As such, both the I.A. injection and I.V. injection routes displayed similar accumulation levels of [^124^I]I-UC-MSC-EVs in the organs, except for the spleen and liver. The higher spleen uptake could importantly provide significant immunomodulatory benefits to UC-MSC-EVs applications for diabetes therapies. These findings may guide the development of safe and efficacious delivery strategies for UC-MSC-EVs.

## 4. Materials and Methods

### 4.1. Chemicals

All reagents were purchased from commercial sources as reagent grade and used without further purification unless otherwise stated. ^124^I-sodium iodide was purchased from 3D Imaging (Little Rock, AR, USA).

### 4.2. Human UC-MSC-EVs Extraction and Purification

Human UC-MSC-EVs were provided from the EVs team at City of Hope using the following steps: (1) isolation of MSCs from human umbilical cord; (2) culture of MSCs in flasks; (3) starvation of MSCs to maximize EV production; and (4) isolation and purification of EVs to obtain UC-MSC-EVs.

### 4.3. Animal

Male Lewis (LEW) rats (Charles River Laboratories, Wilmington, MA, USA) weighing 350–500 g were used to study I.V. and I.A. administration of [^124^I]I-UC-MSC-EVs. Rat thyroid uptake of radioiodine was blocked by pretreatment using 10 drops of saturated KI per 100 mL of drinking water for 24 h before injection of [^124^I]I-UC-MSC-EVs. The use of animals and animal procedures performed in this study were approved by the City of Hope/Beckman Research Institute Institutional Animal Care and Use Committee.

### 4.4. Radioiodination of UC-MSC-EVs and Radiochemical Stability Assessment

Radioiodination of human UC-MSC-EVs with ^124^I-sodium iodide was performed using the direct IODOGEN methodology. Approximately 23 μL of UC-MSC-EVs (2.28 ×10^11^ particles/mL) was added to a tube pre-coated with 150 μg IODOGEN (Pierce, Rockford, IL), followed by 137 MBq [^124^I] NaI diluted to 44 μL in 0.1 M phosphate buffer (pH 7.5) and incubated at room temperature for 3 min. At the end of the incubation, the radioiodinated UC-MSC-EVs were purified by Superdex 200 chromatography (GE Healthcare). The radiochemical purity post-purification was >99% by ITLC. [^124^I]I-UC-MSC-EVs were serviced for animal studies for in vivo evaluation.

The stability of [^124^I]I-UC-MSC-EVs was evaluated for 0, 2, and 4 h. Samples post-incubation were passed to Superoser^®^ 6 10/300 GL column (Running Buffer: 1 × PBS + 0.05% NaN_3_ at 0.4 mL/min) to determine stability.

### 4.5. Procedures for I.V. Administration and Animal PET-CT Imaging

The needle catheter was inserted into a lateral rat tail vein after rats were anesthetized with 2–4% isoflurane in oxygen. Placement of the needle inside the vein was confirmed by infusing a small volume of saline. Rats were transferred into the PET-CT scanner before injection of ~8 MBq [^124^I]I-UC-MSC-EVs. Dynamic microPET scans of 0–90 min were conducted, followed by 1-min CT scans using the small-animal GNEXT PET/CT imaging system (SOFIE, Dulles, VA, USA). The images were reconstructed by three-dimensional ordered subsets expectation-maximization (3D-OSEM) using the integrated GNEXT Acquisition Engine software. Separately, one group of rats underwent I.V. injection as described above, a 90 min dynamic PET scan of the head, and then a 1 min CT scan.

### 4.6. Procedures for I.A. Administration and Animal PET-CT Imaging

All surgical procedures were performed under general anesthesia. To deliver [^124^I]I-UC-MSC-EVs selectively to the pancreatic parenchyma of the body and tail through arterial flow, the following procedures were performed [34]. The abdominal cavity was exposed via a midline abdominal incision through the linea alba of rectus sheath. The descending aorta, celiac trunk, and its main branches of the splenic artery, common hepatic artery, and left gastric artery were identified and exposed (Figure 5A). In addition, small branches of splenic arteries between the pancreatic tail and the spleen were also identified and exposed, since the splenic artery is the dominant feeding artery to pancreatic body and tail as well as spleen. In order to direct the injected solution to the pancreatic body and tail, the arterial flow of the common hepatic artery, left gastric artery, and small branches of splenic arteries between the pancreatic tail and spleen were temporarily blocked using microvascular clamps. (Figure 5A). Approximately 8 MBq [^124^I]I-UC-MSC-EVs were prepared in a 1-mL conventional insulin syringe (using a 25 gauge-needle; BD, Franklin Lakes, NJ, USA) to inject through the celiac trunk. Injection flow was manually controlled at 50 µL/min. After the injection was completed, all clamps were released (duration of arterial clamps were within 5 min), the needle was removed from the celiac trunk, and compression hemostasis was performed with a cotton swab for 5 min. After hemostasis was confirmed, the abdominal incision was closed, and the rat subsequently underwent a 70 min PET-CT scan at 20 min post-injection and then a 1 min CT scan.

For the technical proof of the I.A. surgical procedure, we conducted preliminary tests using 200 µL of Methylene blue dye (diluted with saline at 1:1 dilution [*v/v*] and 0.22 µm-filtered for sterilization) in terminal surgeries. The distribution of the solution in the designated region was confirmed (Figure 5B).

### 4.7. Biodistribution of [^124^I]I-UC-MSC-EVs in Rats

The biodistribution of [^124^I]I-UC-MSC-EVs was investigated at the end of PET-CT scans (96–98 min post injection) for all rats following I.A. and I.V. administration. Rats were anesthetized and euthanized. Blood, heart, lung, liver, spleen, stomach, kidneys, pancreas, small intestine, cecum, large intestine, muscle, fat, bone, and brain were collected and weighed, and the uptake of radioactivity was measured using Hidex AMG Automatic Gamma Counter (HIDEX, Turku, Finland) and its decay was corrected. Results were reported as percentage injected dose per gram (%ID/g).

## 5. Conclusions

In this study, we explored a nuclear imaging technique to monitor and evaluate different delivery modalities for human UC-MSC-EVs in vivo. UC-MSC-EVs were successfully tagged with I-124 via the IODOGEN methodology. Our initial results indicated that UC-MSC-EVs displayed similar delivery efficacy, except in the spleen and liver, for both I.V. and I.A. administration routes in a non-diabetic Lewis (LEW) rat model. Higher uptake in the spleen could, importantly, provide more advantageous immunomodulatory properties for diabetes therapies. As such, these results can guide optimization of UC-MSC-EV delivery strategies for clinical therapies for diabetes. Studies for further confirmation are ongoing.

## Figures and Tables

**Table 1:** Biodistribution of [^124^I]I-UC-MSC-EVs uptake (%ID/gram) *^a^* in the I.V. and I.A. group.

Organ/Tissue	I.V. Group	I.A. Group	*p*-Value
blood	0.41 ± 0.06	0.50 ± 0.02	0.18
heart	0.12 ± 0.01	0.16 ± 0.00	0.06
lung	0.31 ± 0.04	0.33 ± 0.02	0.56
muscle	0.07 ± 0.01	0.13 ± 0.02	0.06
fat	0.02 ± 0.00	0.03 ± 0.00	
kidneys	0.40 ± 0.00	0.39 ± 0.02	0.42
pancreas	0.20 ± 0.06	0.24 ± 0.03	0.47
spleen	1.95 ± 0.03	0.43 ± 0.07	<0.01
small intestine	0.32 ± 0.05	0.42 ± 0.09	0.31
cecum	0.12 ± 0.01	0.20 ± 0.01	0.01
large intestine	0.20 ± 0.04	0.20 ± 0.04	>1.00
stomach	1.05 ± 0.11	0.95 ± 0.07	0.40
liver	0.63 ± 0.21	1.25 ± 0.16	0.08
bone	0.14 ± 0.01	0.14 ± 0.01	0.70
brain	0.03 ± 0.00	0.04 ± 0.01	0.42

*^a^* Data is shown as Mean ± SD.

## Data Availability

All data is contained within article.
